# Pro-Resolving Mediator Annexin A1 Regulates Intracellular Ca^2+^ and Mucin Secretion in Cultured Goblet Cells Suggesting a New Use in Inflammatory Conjunctival Diseases

**DOI:** 10.3389/fimmu.2021.618653

**Published:** 2021-04-22

**Authors:** Anne V. Lyngstadaas, Markus V. Olsen, Jeffrey A. Bair, Robin R. Hodges, Tor P. Utheim, Charles N. Serhan, Darlene A. Dartt

**Affiliations:** ^1^ Schepens Eye Research institute/Massachusetts Eye and Ear, Department of Ophthalmology, Harvard Medical School, Boston, MA, United States; ^2^ Institute of Clinical Medicine, Faculty of Medicine, University of Oslo, Oslo, Norway; ^3^ Department of Medical Biochemistry, Oslo University Hospital, Oslo, Norway; ^4^ Department of Plastic and Reconstructive Surgery, University of Oslo, Oslo, Norway; ^5^ Center for Experimental Therapeutics and Reperfusion Injury, Department of Anesthesia, Brigham and Women’s Hospital and Harvard Medical School, Boston, MA, United States

**Keywords:** ocular surface, annexin A1, secretion, specialized pro resolving mediators, mucin

## Abstract

The amount of mucin secreted by conjunctival goblet cells is regulated to ensure the optimal level for protection of the ocular surface. Under physiological conditions lipid specialized pro-resolving mediators (SPM) are essential for maintaining tissue homeostasis including the conjunctiva. The protein Annexin A1 (AnxA1) can act as an SPM. We used cultured rat conjunctival goblet cells to determine if AnxA1 stimulates an increase in intracellular [Ca^2+^] ([Ca^2+^]_i_) and mucin secretion and to identify the signaling pathways. The increase in [Ca^2+^]_i_ was determined using fura2/AM and mucin secretion was measured using an enzyme-linked lectin assay. AnxA1 stimulated an increase in [Ca^2+^]_i_ and mucin secretion that was blocked by the cell-permeant Ca^2+^ chelator BAPTA/AM and the ALX/FPR2 receptor inhibitor BOC2. AnxA1 increased [Ca^2+^]_i_ to a similar extent as the SPMs lipoxin A_4_ and Resolvin (Rv) D1 and histamine. The AnxA1 increase in [Ca^2+^]_i_ and mucin secretion were inhibited by blocking the phospholipase C (PLC) pathway including PLC, the IP_3_ receptor, the Ca^2+^/ATPase that causes the intracellular Ca^2+^ stores to empty, and blockade of Ca^2+^ influx. Inhibition of protein kinase C (PKC) and Ca^2+^/calmodulin-dependent protein kinase also decreased the AnxA1-stimulated increase in [Ca^2+^]_i_ and mucin secretion. In contrast inhibitors of ERK 1/2, phospholipase A_2_ (PLA_2_), and phospholipase D (PLD) did not alter AnxA1-stimulated increase in [Ca^2+^]_i_, but did inhibit mucin secretion. Activation of protein kinase A did not decrease either the AnxA1-stimulated rise in [Ca^2+^]_i_ or secretion. We conclude that in health, AnxA1 contributes to the mucin layer of the tear film and ocular surface homeostasis by activating the PLC signaling pathway to increase [Ca^2+^]_i_ and stimulate mucin secretion and ERK1/2, PLA_2_, and PLD to stimulate mucin secretion from conjunctival goblet cells.

## Introduction

Inflammation is a component in common to both allergic conjunctivitis and dry eye disease – two frequently occurring diseases of the ocular surface (cornea and conjunctiva) (1). In allergic conjunctivitis allergens penetrate the conjunctival epithelium and initiate the production of pro-inflammatory mediators including histamine, leukotrienes, and prostaglandins. These compounds cause vasodilation, pain, edema and recruitment of neutrophils, macrophages, and mast cells into the conjunctiva (2). In acute inflammation, the infiltrating leukocytes switch from producing pro-inflammatory mediators to generate the specialized pro-resolving lipid mediators (SPMs) lipoxins, resolvins, protectins, and maresins (2–6). These SPMs actively terminate inflammation by blocking the effects of the pro-inflammatory mediators on their target tissues, including the conjunctival goblet cells. The protein annexin A1 (AnxA1) is also pro-resolving (6, 7). In general, failed endogenous resolution mechanisms lead to uncontrolled and chronic inflammation (1). In addition to resolving inflammation, the SPMs also maintain homeostasis of the conjunctival epithelium to keep the ocular surface healthy.

Conjunctival goblet cells synthesize and secrete large, high molecular weight, gel forming mucins (8). These mucins make up the innermost layer of the tear film (9). MUC5AC, the major mucin secreted by conjunctival goblet cells, can trap and remove ocular allergens and airborne pathogens (8). An optimum amount of MUC5AC secreted into the tear film is critical for ocular surface health, as a depleted number of goblet cells and mucin secretion lead to corneal and conjunctival damage (8). Conversely, MUC5AC overproduction is a symptom of allergic conjunctivitis and unhealthy for the ocular surface (8). Lipid SPMs including D-series and E-series resolvins, lipoxins, and maresins act by blocking excess mucin secretion from conjunctival goblet cells during inflammation, as well as stimulating mucin secretion under physiological conditions, thus maintaining ocular surface homeostasis (10–12).

AnxA1 is an anti-inflammatory, pro-resolving protein originally described as a mediator of glucocorticoid action (13). The synthesis of AnxA1 is a primary anti-inflammatory mechanism of glucocorticoids. AnxA1 works at least partly by suppressing phospholipase A_2_ activity, thus blocking the production of pro-inflammatory eicosanoids (14). During inflammation, endogenous glucocorticoids are secreted from the adrenal gland to avoid an excessively vigorous inflammatory response that could potentially damage the host (15). Glucocorticoids can stimulate both the synthesis and secretion of AnxA1 (16). After being synthesized AnxA1 is mobilized to the plasma membrane and secreted by three different mechanisms: 1) the ATP-binding (ABC) transporter, 2) direct phosphorylation of serine-27 that localizes it to the plasma membrane, and 3) exocytosis by granule fusion with the cell membrane (17). The secreted AnxA1 can then interact with the ALX/FPR2 receptor in a paracrine, autocrine, or juxtacrine fashion.

As an effector molecule of glucocorticoids, AnxA1 has potential as an endogenous anti-inflammatory drug, and the use of AnxA1 therapeutically likely has fewer side effects than the use of glucocorticoids. AnxA1 contributes to the regulation of a wide variety of cellular events, including both acute and chronic inflammation, ischemic injury, fever, pain, and the release of arachidonic acid (14). AnxA1, as well as its N-terminal peptides, have also been found to potently inhibit neutrophil trafficking in mice (7). AnxA1 could prevent development of pro-inflammatory disease in the conjunctiva.

To determine if AnxA1 plays a role in maintaining the mucous layer in ocular surface health we investigated if exogenous addition of AnxA1 can increase intracellular [Ca^2+^] and stimulate MUC5AC secretion in cultured primary conjunctival goblet cells from rats and determined which signaling pathways AnxA1 activates in these cells.

## Materials and Methods

### Materials

AnxA1 was obtained from MyBiosource (San Diego, CA). The compound was stored at -20°C. Prior to use, AnxA1 was immediately diluted in Krebs-Ringer bicarbonate buffer with HEPES (KRB-HEPES, 119 mM NaCl, 4.8 mM KCl, 1.0 mM CaCl_2_, 1.2 mM MgSO_4_, 1.2 mM KH_2_PO_4_, 25 mM NaHCO_3_, 10 mM HEPES, and 5.5 mM glucose [pH 7.45]) to the required concentration and added to the conjunctival goblet cells. RPMI-1640 cell culture medium, penicillin/streptomycin, trypsin, and L-glutamine were purchased from Lonza (Walkerville, IL). Fetal bovine serum (FBS) was from Atlanta Biologicals (Norcross, GA). Fura-2 was from Life Technologies (Grand Island, NY). U73122 and U73343, KN92 and KN93 were purchased from Tocris Bioscience (Ellisville, MO). The lectin Ulex Europaeus Agglutinin (UEA-1), histamine, BAPTA/AM, 2-aminoethyl diphenylborinate (2-APB), 1-butanol (1-but), *t*-butanol (*t*-but), carbachol, aristolochic acid (AA), thapsigargin and RO317549 were all purchased from Sigma-Aldrich (St Louis, MO). UO126 was obtained from R&D Systems (Minneapolis, MN). H89 and synthetic lipoxin A_4_ (LXA_4_, 5(S),6(R),15(S)-TriHETE) were from EMD Millipore (Billerica, MA). Resolvin (Rv) D1 (7S, 8R, 17S-trihydroxy-4Z, 9E 11E, 13Z, 19Z-docosahexaenoic acid) was purchased from Cayman Chemical, Ann Arbor, MI). N-Boc-Phe-Leu-Phe-Leu-Phe (BOC2) was obtained from Genscript Corp in Piscataway, NJ.

### Animals

Four- to eight-week-old male Sprague-Dawley rats (Taconic Farms, Germantown, NY, USA) were anesthetized with CO_2_ and decapitated. The bulbar and forniceal conjunctival epithelium was surgically removed from both eyes. All experiments were in accordance with the US National Research Council’s Guide for the Care and Use of Laboratory Animals, the US Public Health Service’s Policy on Humane Care and Use of Laboratory Animals, and Guide for the Care and Use of Laboratory Animals and were approved by the Schepens Eye Research Institute Animal Care and Use of Committee.

### Culture of Conjunctival Goblet Cells

Goblet cells from rat conjunctiva were grown in organ culture as described previously (3). The conjunctiva was dissected free of underlying connective tissue. The conjunctival explants were grown on six-well plates in RPMI 1640 medium supplemented with 10% fetal bovine serum (FBS), 2 mM glutamine, and 100 μg/mL penicillin-streptomycin for approximately one week. The goblet cells were then trypsinized and plated onto glass bottom petri dishes or 24 well plates, for [Ca^2+^]_i_ measurements or mucin secretion measurements, respectively. First passage goblet cells were used in all experiments. The identity of the cultured cells was checked by evaluating staining with antibody to cytokeratin 7 (detects goblet cell bodies) and the lectin UEA-1 (detects goblet cell secretory products) to ensure that goblet cells predominated in the cultures.

### Measurement of cDNA Expression by Semi-Quantitative PCR

Cultured goblet cells were homogenized in TRIzol and total RNA isolated according to manufacturer’s instructions. One microgram of total RNA was used for complementary DNA (cDNA) synthesis using the Superscript First-Strand Synthesis system for RT-PCR (Invitrogen, Carlsbad, CA). The cDNA was amplified by the polymerase chain reaction (PCR) using primers specific to AnxA1 using the Jumpstart REDTaq Readymix Reaction Mix (Sigma-Aldrich, St. Louis, MO) in a thermal cycler (Master Cycler, Eppendorf, Hauppauge, NY). The forward primer was GTG ATC GCT GTG AGG ATA TGA G, and the reverse primer was TAC AGA GCA GTT GGG ATG TTT AG. These primers generated 504 bp fragments. β-Actin primers, the positive control, were forward CGT CAT ACT CCT GCT TGC TGA TCC A and the reverse primer was ATC TGG CAC CAC ACC TTC TAC AAT GG CT and generated a 790 bp fragment. The conditions were as follows: 5 min at 95°C followed by 35 cycles of 1 min at 94°C, 30 seconds at annealing temperature for 1 min at 72°C with a final hold at 72°C for 10 min. Samples with no cDNA served as the negative control. Amplification products were separated by electrophoresis on a 1.5% agarose gel and visualized by ethidium bromide staining.

### Measurement of Mucin Secretion

Cultured rat goblet cells were plated in 24-well plates and grown to confluence (1, 10–12, 18, 19). After serum starving cultured goblet cells for 2 h, the goblet cells were stimulated with AnxA1 in increasing concentrations in serum-free RPMI 1640 supplemented with 0.5% BSA for 2 h. The inhibitors were added 30 minutes prior to stimulation with AnxA1. An enzyme-linked lectin assay (ELLA) with the lectin UEA-1 was used to measure high molecular weight glycoprotein secretion that included MUC5AC produced by goblet cells. The supernatants were collected and analyzed for the total amount of lectin-detectable glycoconjugates, which quantifies the amount of goblet cell secretion as described previously (1, 3, 10–12, 20–22). The cells were scraped, and the cell homogenate was analyzed for the total amount of protein by using the Bradford protein assay. High molecular weight glycoconjugate secretion was normalized to total protein in the homogenate. Secretion was expressed as fold increase above basal that was set to 1.

### Measurement of Intracellular [Ca^2+^]

Cultured rat conjunctival goblet cells were transferred to 35-mm glass bottom culture dishes and incubated overnight. The goblet cells were then incubated for 1 h at 37°C with Krebs-Ringer bicarbonate buffer with HEPES (KRB-HEPES) (119 mM NaCl, 4.8 mM KCl, 1.0 mM CaCl_2_, 1.2 mM MgSO_4_, 1.2 mM KH_2_PO_4_, 25 MM NaHCO_3_, 10 mM HEPES, and 5.5 mM glucose) (pH 7.45) plus 0.5% BSA containing 0.5 fura-2/AM (Invitrogen, Grand Island, NY), 8 μM pluronic acid F127 (Sigma-Aldrich, St. Louis, MO, USA) and 250 μM sulfinpyrazone (Sigma-Aldrich), followed by washing in KRB-HEPES containing sulfinpyrazone. Ca^2+^ measurements were made with a ratio imaging system (InCyt Im2; Intracellular Imaging, Cincinnati, OH) using excitation wavelengths of 340 and 380 nm and an emission wavelength of 505 nm. A minimum of 10 cells were selected in each experimental condition. AnxA1 was added either alone or after incubation with inhibitors for 15 or 30 min and intracellular [Ca^2+^] ([Ca^2+^]_i_) measured. After addition of agonist, data were collected in real time. The data are presented as [Ca^2+^]_i_ over time and peak [Ca^2+^]_i_, calculated by subtracting the average value before the agonist AnxA1 was added (basal) from the peak [Ca^2+^]_i_.

### Statistical Analysis

Results were expressed as the fold-increase above basal. Data are expressed as mean ± SEM. Data were analyzed by Student’s t-test. p< 0.05 was considered statistically significant.

## Results

### AnxA1 Is Expressed in Rat Conjunctival Goblet Cells

**Figure 1:**
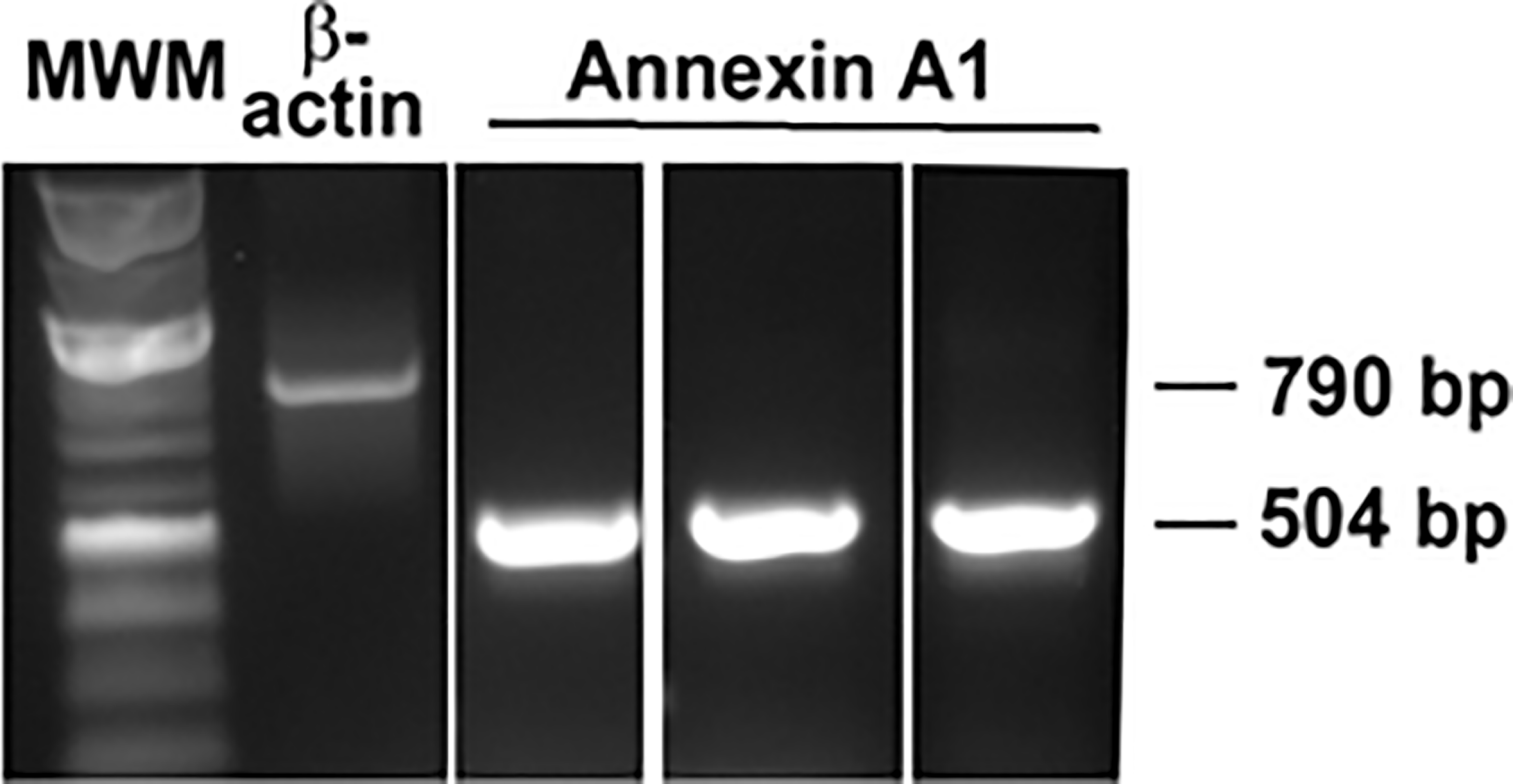
AnxA1 expression is detected in cultured rat conjunctival goblet cells by RT-PCR. Each lane represents cDNA from a different animal. β-Actin was used as a control.

The protein AnxA1 was detected in a wide range of tissues, including the lung, intestine and bone marrow (14). We used PCR to investigate the expression of AnxA1 in cultured rat conjunctival goblet cells (**Figure 1**). A single band at the correct size of 504 bp was found in cDNA isolated from the conjunctivas of three individual animals. Expression of β-actin was used as a control. As AnxA1 is a protein, this is the first pro-resolving mediator whose presence in rat conjunctival goblet cells was verified by PCR.

### AnxA1 Stimulates Mucin Secretion in Rat Conjunctival Goblet Cells

**Figure 2:**
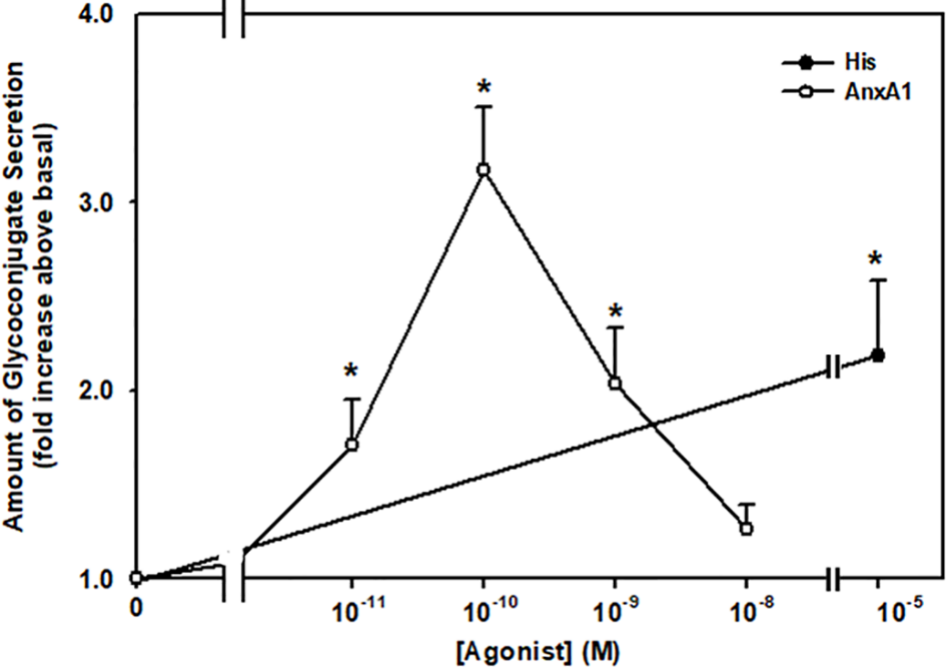
AnxA1 increases glycoconjugate secretion from cultured rat conjunctival goblet cells. Goblet cells were incubated with increasing concentrations of annexin A1 (AnxA1, 10^-11^ - 10^-8^ M) or histamine (His, 10^-5^ M). Data are presented as mean ± SEM from three independent experiments. * Indicates significant difference from basal.

Lipid specialized pro-resolving mediators (SPM) such as LXA_4_, RvD1, and RvE1 stimulate mucin secretion from cultured rat conjunctival goblet cells (10–12). To explore if the protein AnxA1 stimulates secretion, goblet cells were stimulated for two h with AnxA1 (10^-11^-10^-8^ M) or a positive control histamine (10^-5^ M) (18, 23) and the amount of secretion was then measured (**Figure 2**). Histamine is known to stimulate glycoconjugate secretion from conjunctival goblet cells (22, 24). AnxA1 increased mucin secretion in a concentration-dependent manner with a maximum increase at 10^-10^ M. Significant increases occurred from 10^-11^ M to 10^-9^ M AnxA1 and were 1.7 ± 0.2, 3.2 ± 0.3 and 2.0 ± 0 fold above basal, respectively from three independent experiments. In comparison histamine significantly increased mucin secretion from basal by 2.2 ± 0.4 fold.

### AnxA1 Increases [Ca^2+^]i in Rat Conjunctival Goblet Cells

**Figure 3:**
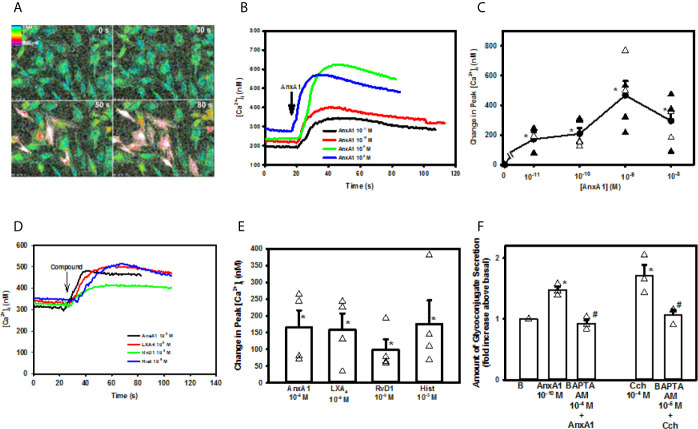
AnxA1 elevates [Ca^2+^]_i_ to stimulate glycoconjugate secretion in cultured rat conjunctival goblet cells. Cultured goblet cells were incubated with fura-2/AM and stimulated with increasing concentrations of Annexin A1 (AnxA1, 10^-11^ M to 10^-8^ M). Pseudo color images of [Ca^2+^]_i_ of goblet cells stimulated by AnxA1 10^-9^ M at four times are shown in **(A)** The increase in [Ca^2+^]_i_ over time with AnxA1 (10^-11^ M to 10^-8^ M) is shown in **(B)** The change in peak [Ca^2+^]_i_ after stimulation with AnxA1 (10^-11^ M to 10^-8^ M) is shown in **(C)** Increase in [Ca^2+^]_i_ over time in response to AnxA1 (10^-9^ M), LXA_4_ (10^-9^ M), RvD1 (10^-9^ M) and histamine (hist, 10^-5^ M) is shown in **(D)** The change in peak [Ca^2+^]_i_ with AnxA1, LXA_4_, RvD1, and histamine is shown in **(E)** Glycoconjugate secretion from goblet cells treated vehicle, AnxA1 (10^-10^ M), with the Ca^2+^ chelator BAPTA-AM (10^-4^ M) followed by stimulation with AnxA1, carbachol (Cch, 10^-4^ M) or BAPTA-AM (10^-4^ M) followed by carbachol is shown in **(F)** Data are mean ± SEM from 4 independent experiments **(A–E)** or 3 **(F)** independent experiments. *indicates significant difference from basal; ^#^indicates significant difference from AnxA1 or Cch alone.

As AnxA1 stimulates conjunctival goblet cells to secrete mucins, we determined if AnxA1 alters the [Ca^2+^]_i_. Cultured goblet cells were incubated with fura-2/AM before stimulation with AnxA1 (10^−11^ -10^−8^ M). AnxA1 increased [Ca^2+^]_i_ in a time and concentration-dependent manner (**Figures 3A–C**). All concentrations of AnxA1 significantly increased [Ca^2+^]_i_ with the highest increase of 467.3 ± 95.1 nM occurring at 10^-9^ M (**Figure 3C**). These data are from five independent experiments. Therefore, AnxA1 increased [Ca^2+^]_i_ in conjunctival goblet cells.

To compare the [Ca^2+^]_i_ responses induced by other known mediators with that of AnxA1, goblet cells were stimulated with AnxA1, the lipid SPMs LXA_4_ and RvD1, and the allergic mediator histamine. All compounds were used at the concentrations previously found to result in the highest increase in [Ca^2+^]_i_ (10, 12). Each mediator significantly increased [Ca^2+^]_i_ over basal and to the same extent, as there was no significant difference between them (n=4, **Figures 3D, E**).

### AnxA1 Uses Intracellular Ca^2+^ to Stimulate Secretion in Rat Conjunctival Goblet Cells

To determine if the AnxA1-stimulated increase in [Ca^2+^]_i_ leads to mucin secretion, rat goblet cells were incubated with the intracellular calcium chelator BAPTA/AM (10^-4^ M) for 30 minutes prior to AnxA1 (10^-9^ M) stimulation. The cholinergic agonist carbachol (Cch) at 10^-4^ M was used as a control. To ensure that BAPTA chelated [Ca^2+^]_i_ and prevented the increase in [Ca^2+^]_i_ induced by AnxA1 and Cch, [Ca^2+^]_i_ was measured (**Supplemental Figure 1**). AnxA1 significantly increased [Ca^2+^]_i_ to a peak of 128.8 ± 11.5 nM.When goblet cells were preincubated with BAPTA/AM, the AnxA1-stimulated increase in [Ca^2+^]_i_ was significantly inhibited to 30.1 ± 7.5 nM. The Ca^2+^ response to Cch, a positive control, was also significantly inhibited from 227.0 ± 54.8 nM to 29.3 ± 50.2 nM in the presence of BAPTA. These results were from 4 independent experiments.

We then investigated if AnxA1 uses $\text{C}{\text{a}}_{i}^{2+}$ to increase mucin secretion. Rat cultured goblet cells were treated with BAPTA/AM (10^-4^ M), stimulated by AnxA1 (10^-10^ M) and secretion measured. In three independent experiments, AnxA1 alone significantly increased glycoconjugate secretion by 1.5 ± 0.05 fold above basal (**Figure 3F**). BAPTA/AM significantly inhibited AnxA1-stimulated mucin secretion to 0.9 ± 0.06 fold above basal. Cch (10^-4^ M)-stimulated glycoconjugate secretion was also significantly inhibited from 1.7 ± 0.2 fold to 1.1 ± 0.1 fold above basal. These findings demonstrate that AnxA1 increases [Ca^2+^]_i_ to stimulate mucin secretion in conjunctival goblet cells.

### AnxA1 Uses Both Intracellular Ca^2+^ Stores and Extracellular Ca^2+^ to Increase [Ca^2+^]_i_ in Rat Conjunctival Goblet Cells

**Figure 4:**
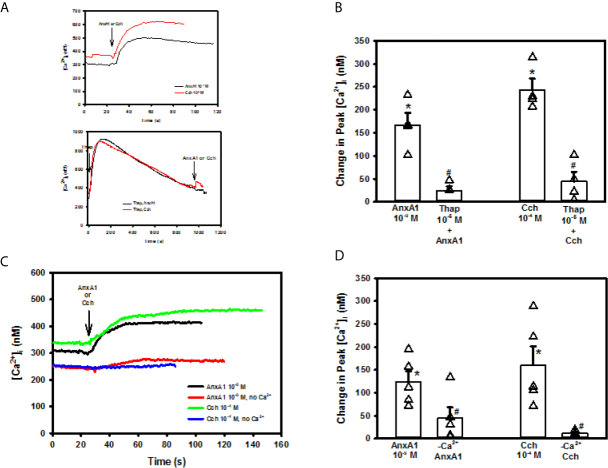
Annexin A1 (Anx1, 10-9 M) or carbachol (Cch, 10-4 M) (A top panel) or AnxA1 uses intracellular and extracellular Ca^2+^ to increase [Ca^2+^]_i_ in cultured rat conjunctival goblet cells. Cultured rat goblet cells were treated with thapsigargin (10^-5^ M) (first arrow) for 15 minutes before stimulation indicated by second arrow with annexin A1 (AnxA1, 10^-9^ M) or Cch (10^-4^ M). (A bottom panel), [Ca^2+^]_i_ over time is shown in **(A)** Change in peak [Ca^2+^]_i_ is shown in **(B)** Goblet cells were incubated in KRB solution with and without CaCl_2_ and stimulated with AnxA1 (10^−9^ M) or Carbachol (Cch, 10^-4^ M). [Ca^2+^]_i_ over time is shown in **(C)** Change in peak [Ca^2+^]_i_ is shown in **(D)** Data are mean ± SEM from 4 **(A, B)** or 6 **(C, D)** independent experiments. *indicates significant difference from basal; ^#^indicates significance from AnxA1 or Cch alone.

To examine the role that intracellular Ca^2+^ stores play in AnxA1 stimulation, goblet cells were incubated with the Ca^2+^ reuptake inhibitor thapsigargin. Thapsigargin depletes the ER of Ca^2+^ by blocking the Ca^2+^/ATPase in the endoplasmic reticulum (ER) that inhibits Ca^2+^ from being taken up by the ER (25). The ER store of Ca^2+^ is depleted as Ca^2+^ passively leaks out of the ER and is not replaced as shown by the increase in [Ca^2+^]_i_ over time (**Figure 4A**). AnxA1 added alone induced a peak increase in [Ca^2+^]_i_ of 166.8 ± 26.7 nM (**Figures 4A, B**, n=6). The AnxA1-induced Ca^2+^ response was significantly blocked by thapsigargin pre-treatment and was 24.6 ± 9.7 nM. In the same cells, Cch was used as a control and increased [Ca^2+^]_i_ to 243.4 ± 24.0 nM. Cch-stimulated increase in [Ca^2+^]_i_ was also blocked by pre-treatment with thapsigargin and was 44.4 ± 21.2 nM. These experiments indicate that AnxA1 works by mobilizing Ca^2+^ from the ER to increase cytoplasmic [Ca^2+^]_i_.

After an agonist depletes intracellular Ca^2+^ stores, this depletion activates STIM1 and Orai to induce Ca^2+^ influx to replace those stores. Since AnxA1 used intracellular Ca^2+^ stores, we investigated if AnxA1 used extracellular Ca^2+^. Cells were incubated in KRB with and without extracellular CaCl_2_. AnxA1 (10^-9^ M) added to the KRB containing CaCl_2_ significantly increased [Ca^2+^]_i_ by 124.1 ± 23.0 nM (**Figures 4C, D**, n=4). This increase in [Ca^2+^]_i_ was significantly reduced to 44.0 ± 23.7 nM when AnxA1 was added to KRB without CaCl_2_, indicating that AnxA1 stimulates influx of extracellular Ca^2+^ to increase [Ca^2+^]_i_. Cch (10^-4^ M), a control, elevated [Ca^2+^]_i_ to 160.4 ± 40.9 nM. Cch-stimulated increase in [Ca^2+^]_i_ was significantly blocked by the CaCl_2_-free solution and was 11.8 ± 2.1 nM. Based on these results, AnxA1 releases Ca^2+^ from intracellular Ca^2+^ stores and stimulates extracellular Ca^2+^ influx to increase the [Ca^2+^]_i_ in conjunctival goblet cells.

### AnxA1 Uses the ALX/FPR2 Receptor to Increase [Ca^2+^]_i_ and Stimulate Secretion in Rat Conjunctival Goblet Cells

**Figure 5:**
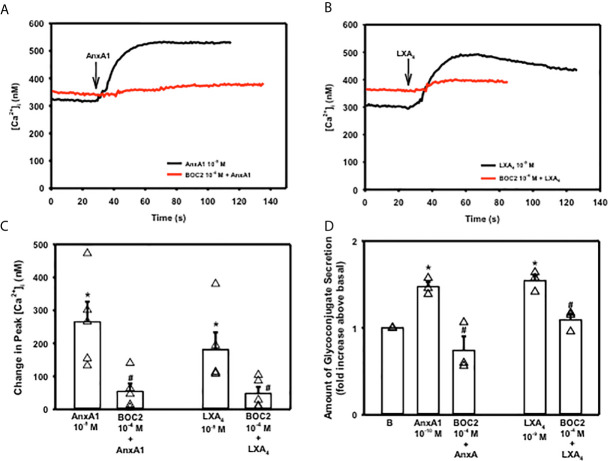
AnxA1 uses the ALX/FPR2 receptor to increase [Ca^2+^]_i_ and stimulate secretion in cultured rat conjunctival goblet cells. Goblet cells were preincubated with BOC2 (10^-4^ M) for 30 minutes and stimulated with vehicle or annexin A1 (AnxA1, 10^-9^ M) shown in **(A, C)** and vehicle or the control LXA_4_ (10^-9^ M) shown in **(B, C)** [Ca^2+^]_i_ over time is shown in **(A, B)** Change in peak [Ca^2+^]_i_ is shown in **(C)** Amount of glycoconjugate secretion is shown in **(D)** with AnxA1 at 10^-10^ M. B indicates basal (vehicle) in **(D)** Data in are mean ± SEM from 4 **(A–C)** or 3 **(D)** independent experiments. *indicates significant difference from basal; ^#^indicates significance from AnxA1 or LXA_4_ alone.

In tissues and cells examined, AnxA1 works through the G-protein coupled formyl peptide receptor 2 (ALX/FPR2) (14, 26). To confirm that AnxA1 uses this receptor in goblet cells, cultured rat conjunctival goblet cells were incubated with the ALX/FPR2 antagonist BOC-2 (10^-4^ M) for 30 minutes before stimulating the goblet cells with AnxA1 (10^-9^ M) or LXA_4_ (10^-9^ M), which is known to bind to ALX/FPR2 and is a positive control (12). AnxA1 added alone significantly increased [Ca^2+^]_i_ with a peak of 265.4 ± 61.0 nM (**Figures 5A, C**, n=5). BOC-2 significantly blocked AnxA1’s increase in [Ca^2+^]_i_ to 54.1 ± 23.9 nM. The LXA_4_ response was 180.8 ± 52.4 nM (**Figures 5B, C**). In the presence of BOC-2, the LXA_4_ response was significantly reduced to 47.7 ± 20.0 nM.

To determine if AnxA1 uses the ALX/FPR2 receptor to stimulate high molecular weight glycoprotein secretion, cells were preincubated with BOC-2 (10^-4^ M) followed by AnxA1 (10^-10^ M) (**Figure 5D**, n=3). LXA_4_ (10^-9^ M) was again used as a positive control. Secretion was significantly increased by 1.5 ± 0.05 fold above basal when AnxA1 (10^-10^ M) was added by itself. AnxA1 stimulated secretion was significantly inhibited by BOC-2 to 0.74 ± 0.16 fold above basal. LXA_4_ stimulated secretion was also inhibited by BOC-2. These data indicate that AnxA1 uses the ALX/FPR2 receptor to stimulate an increase in [Ca^2+^]_i_ and secretion in conjunctival goblet cells.

### AnxA1 Activates the PLC Pathway and Its Downstream Effectors IP_3_ and PKC to Increase [Ca^2+^]_i_ and Stimulate Secretion in Rat Conjunctival Goblet Cells

**Figure 6:**
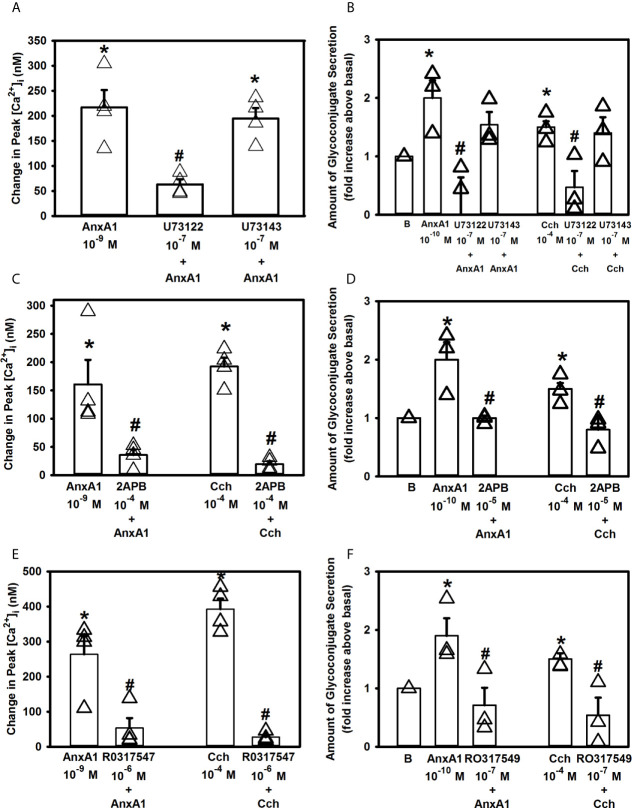
AnxA1 activates the Phospholipase C (PLC) pathway and its downstream effectors to increase [Ca^2+^]_i_ in cultured rat conjunctival goblet cells. Cultured goblet cells were treated with vehicle, the PLC inhibitor U73122, its inactive analog U73343 (both at 10^-7^ M, **A**, **B**), 2APB (10^-5^ M, **C**, **D**), or the PKC inhibitor RO317549 (10^-7^ M, **E**, **F**) for 30 minutes before stimulation with annexin A1 (AnxA1, 10^-9^ M) in **(A, C, E)** or with AnxA1 at 10^-10^ M in **(B, D, F)** or the positive control carbachol (Cch, 10^-4^). Change in peak [Ca^2+^]_i_ are shown in **(A, C, E)** while glycoconjugate secretion is shown in **(B, D, F)**. B (vehicle) indicates basal in **(B, D, F)**. Data are mean ± SEM from three **(A, B, D, F)**, four **(C, E)** independent experiments. *indicates significant difference from basal; ^#^indicates significance from AnxA1 or carbachol alone.

The phospholipase C (PLC) pathway is critical for cellular Ca^2+^ signaling as activation of PLC generates both inositol 1,4,5-trisphosphate (IP_3_) and diacylglycerol (DAG) (27). IP_3_ interacts with its receptors on the ER to release Ca^2+^ from intracellular stores, while DAG activates protein kinase C (PKC). To explore if AnxA1 activates the PLC pathway in rat conjunctival goblet cells, these cells were preincubated with either the PLC inhibitor U73122 or its negative control U73343 at 10^-7^ M, before addition of AnxA1 (10^-9^ M) or a positive control Cch [10^-4^ M) (28)]. AnxA1 added alone significantly increased [Ca^2+^]_i_ with a peak of 217.1 ± 34.6 nM (**Figure 6A**, n=3) The active inhibitor U73122 significantly decreased the AnxA1 stimulated increase in [Ca^2+^]_i_ to 63 ± 10.0 nM, while the inactive inhibitor U73343 did not significantly block AnxA1. The Cch response was also inhibited by U73122, but not U73343 (28).

The effects of U73122 and U73143 on secretion were then determined. AnxA1 (10^-10^ M) increased secretion 2.0 ± 0.3 fold above basal (**Figure 6B**, n=3). AnxA1-stimulated response was completely inhibited by preincubation with U73122 (10^-7^ M), but not U73143 (10^-7^ M). U73122, but not U73143, also significantly inhibited secretion stimulated by Cch (10^-4^ M), which was a positive control. These data show that AnxA1 stimulates PLC to increase [Ca^2+^]_i_ and stimulate secretion.

To further explore the PLC pathway, goblet cells were incubated for 30 min with 2APB (10^-5^ M), an inhibitor of IP_3_ receptors on the ER (29). AnxA1 (10^-9^ M) added alone significantly increased [Ca^2+^]_i_ by 160.6 ± 43.4 nM (**Figure 6C**, n=4). AnxA1-induced [Ca^2+^]_i_ increase was significantly decreased to 35.7 ± 9.6 nM after 30 minutes of incubation with 2APB. Cch (10^-4^ M) was used as a positive control and its stimulation of [Ca^2+^]_i_ was significantly blocked by 2APB as well.

A similar effect of 2APB was found when secretion was examined. AnxA1 (10^-10^ M) increased secretion 2.0 ± 0.3 fold above basal (**Figure 6D**, n=3). AnxA1-stimulated response was significantly inhibited by incubation with 2APB, which also significantly inhibited secretion stimulated by Cch (10^-4^ M), the positive control.

Next, we investigated the effect of the PKC inhibitor RO317549. Goblet cells were incubated for 30 minutes with RO317549 (10^-7^ M) before stimulation with AnxA1 (10^-9^ M) or a positive control Cch (10^-4^ M). [Ca^2+^]_i_ was significantly increased when AnxA1 was added alone (263.7 ± 51.8 nM, **Figure 6E**, n=4). RO317549 significantly blocked the effect of AnxA1 on [Ca^2+^]_i_ and was 27.0 ± 6.3 nM)). Cch-stimulated increase in [Ca^2+^]_i_ was also significantly inhibited.

Inhibition of PKC with RO317549 also blocked AnxA1-stimulated secretion. AnxA1 increased secretion by 1.9 ± 0.3 fold above basal (**Figure 6F**, n=3). RO317549 significantly reduced AnxA1-induced secretion to 0.7 ± 0.3 fold above basal. As a positive control, Cch stimulated secretion was also significantly inhibited

In conjunctival goblet cells inhibitors of PLC activity, IP_3_ interaction with its receptors, and PKC each blocked the AnxA1-stimulated function measured. Based on these findings, AnxA1 activates the PLC pathway both to increase intracellular Ca^2+^ and stimulate secretion through the production of IP_3_ and activation of PKC.

### AnxA1 Increases [Ca^2+^]_i_ and Secretion Through Activation of Ca^2+^/CaMK II in Conjunctival Goblet Cells

**Figure 7:**
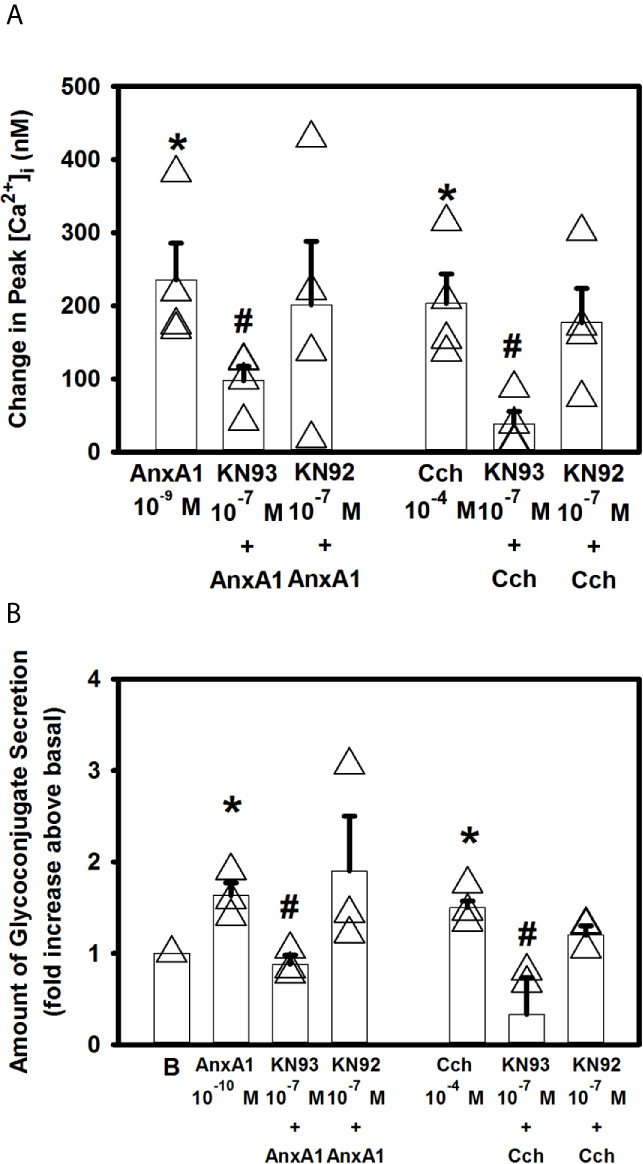
AnxA1 activates the calcium/calmodulin-dependent protein kinase (Ca^2+^/CaMK) to increase [Ca^2+^]_i_ and stimulate glycoconjugate secretion in cultured rat conjunctival goblet cells. Goblet cells were treated with the Ca^2+^/CaMK inhibitor KN93, its inactive analog KN92,or vehicle for 30 minutes prior to stimulation with annexin A1 (AnxA1, 10^-9^ M) or carbachol (Cch, 10^-4^ M). Change in peak [Ca^2+^]_i_ is shown in **(A)** AnxA1 (10^-10^ M)-stimulated glycoconjugate secretion is shown in **(B)** indicates basal (vehicle) in **(B)** Data are mean ± SEM from 4 **(A)** or 3 **(B)** independent experiments. *indicates significant difference from basal. ^#^indicates significance from Cch alone.

Free intracellular Ca^2+^ can bind to calmodulin, to create a calmodulin/calcium complex. This complex can bind to different proteins such as Ca^2+^/calmodulin dependent protein kinase II (Ca^2+^/CaMK) (30). We have previously shown that LXA_4_, through the ALX/FPR2 receptor, increases [Ca^2+^]_i_ through Ca^2+^/CaMK (12). To investigate if AnxA1 also activates Ca^2+^/CaMK, goblet cells were preincubated with the Ca^2+^/CaMK inhibitor KN93 or the inactive analog KN92 at 10^-7^ M for 30 minutes. AnxA1 (10^-9^ M) significantly increased [Ca^2+^]_i_ to a peak of 235.6 ± 50.0 nM (**Figure 7A**). KN93 but not KN92 significantly blocked AnxA1-stimulated increase in [Ca^2+^]_i_ and was 97.6 ± 19.6 (**Figure 7A**, n=4). A positive control Cch (10^-4^ M) significantly increased [Ca^2+^]_i_ by 203.5 ± 40.2 nM. KN 93, but not KN92, significantly decreased Cch-stimulated increase in [Ca^2+^]_i_.

KN93 also significantly decreased 10^-10^ M AnxA1-stimulated secretion from 1.6 ± 0.1 to 0.9 ± 0.1 fold above basal (**Figure 7B**, n=3). KN92 had no effect on AnxA1-stimulated secretion. KN93, but not KN92, also significantly inhibited 10^-4^ M Cch-stimulated secretion. These data indicate that AnaX1 uses Ca^2+^/CaMK to increase [Ca^2+^]_i_ and stimulate glycoconjugate secretion.

### AnxA1 Does Not Increase [Ca^2+^]_i_ But Does Increase Secretion Through Activation of ERK 1/2 in Rat Conjunctival Goblet Cells

**Figure 8:**
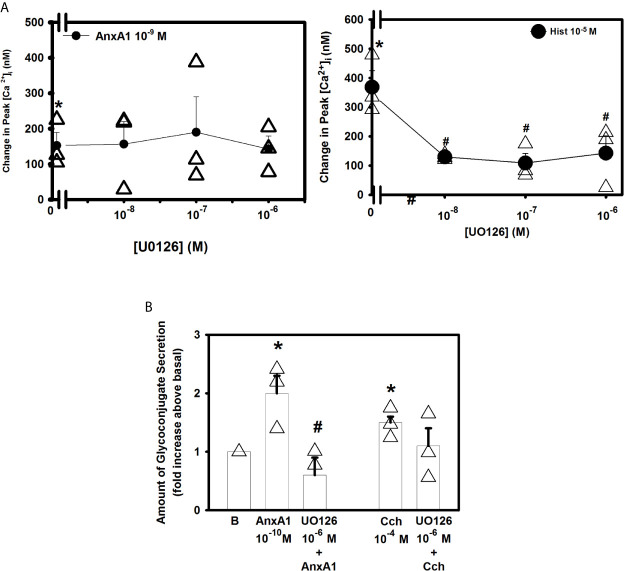
AnxA1 does not activate extracellular regulated protein kinase (ERK) 1/2 to increase [Ca^2+^]_i_ but does activate it to stimulate glycoconjugate secretion in cultured rat conjunctival goblet cells. Rat conjunctival goblet cells were treated with vehicle or the ERK 1/2 inhibitor UO126 (10^-8^ - 10^-6^ M) for 30 minutes prior to stimulation with either annexin A1 (AnxA1, 10^-9^ M) in **(A)** or with AnxA1 at 10^-10^ M in **(B)**, or the control histamine (Hist, 10^-5^ M) or carbachol (Cch, 10^-4^ M). Change in peak [Ca^2+^]_I_ is shown in **(A)** Glycoconjugate secretion is shown in **(B)** B indicates basal (vehicle) in **(B)** Data are mean ± SEM from 4 **(A)** or 3 **(B)** independent experiments. *indicates significant difference from basal. ^#^indicates significance from either AnxA1 or histamine alone.

The ERK 1/2 pathway is a component of a signaling cascade that is activated in cells by a variety of stimuli (18, 31, 32). To determine if the ERK 1/2 pathway plays a role in AnxA1-stimualted increase in [Ca^2+^]_i_ the ERK 1/2 inhibitor UO126 was used. Rat conjunctival goblet cells were incubated with UO126 (10^-8^–10^-6^ M) for 30 minutes (**Figure 8A**, n=3). AnxA1 (10^-9^ M) added alone significantly increased [Ca^2+^]_i_ by 152.5 ± 37.2 nM. AnxA1-induced [Ca^2+^]_i_ increase was not significantly blocked by UO126 at any concentration. Histamine (10^-5^ M), which is known to activate ERK 1/2 was used as a control (22) and was significantly blocked by all three concentrations of UO126.

To examine the effects of UO126 on glycoconjugate secretion, goblet cells were incubated with AnxA1 with and without UO126 (10^-7^ M). AnxA1(10^-10^ M) alone increased secretion by 2.0 ± 0.3 fold above basal (**Figure 8B**, n=3). UO126 significantly decreased AnxA1-stimulated response and was 0.6 ± 0.3 fold above basal. In contrast to previous studies (31) U0126 did not inhibit the carbachol-induced secretory response. These data indicate that AnxA1 does not activate ERK1/2 to increase [Ca^2+^]_i_ but does activate it to stimulate glycoconjugate secretion.

### AnxA1 Does Not Use PKA to Increase [Ca^2+^]_i_ in Rat Conjunctival Goblet Cells

To determine if AnxA1 increases cAMP levels to activate protein kinase A (PKA) to increase [Ca^2+^]_i_ and stimulate secretion, goblet cells were preincubated with the PKA inhibitor H89 (10^-5^ M) for 30 minutes before stimulating the cells with AnxA1 (10^-9^ M) (**Supplemental Figure 2A**, n=9). AnxA1 added alone significantly increased [Ca^2+^]_i_ by 420 ± 111.9 nM. H89 did not significantly inhibit AnxA1 stimulated increase in [Ca^2+^]_i_. Vasoactive intestinal peptide (VIP, 10^-8^ M), which is known to increase cAMP (19), was used as a control. VIP-stimulated increase in [Ca^2+^]_i_ was 548.1 ± 110.6 nM. H89 significantly blocked VIP-stimulated increase in [Ca^2+^]_i_ and was 252.5 ± 39.6 nM.

Similar to the effects on [Ca^2+^]_i_ H89 did not significantly inhibit AnxA1-stimulated glycoconjugate secretion (**Supplemental Figure 2B**, n=3). However, the positive control, VIP-stimulated secretion was inhibited by H89. These data indicate that activation of PKA does not play a role in AnxA1-stimulated increase in [Ca^2+^]_i_ or secretion.

### AnxA1 Does Not Increase [Ca^2+^]_i_ But Does Stimulate Secretion Through the PLD Pathway in Rat Conjunctival Goblet Cells

**Figure 9:**
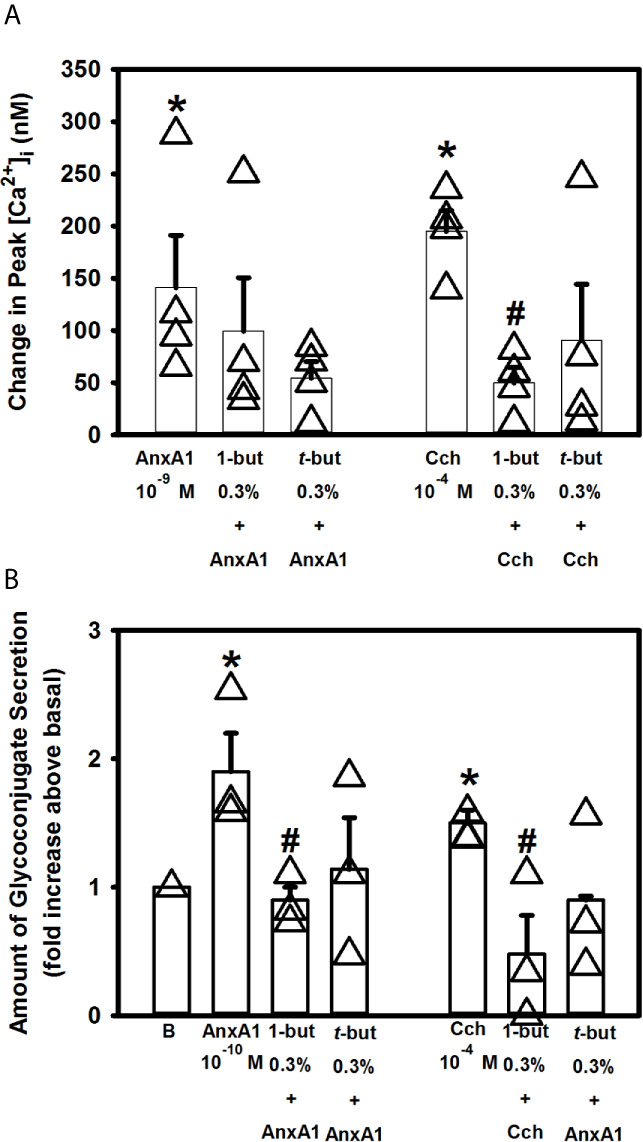
AnxA1 does not active the phospholipase D (PLD) pathway to increase [Ca^2+^]_i_ but activates it to stimulate secretion in cultured rat conjunctival goblet cells. Rat conjunctival goblet cells were treated with vehicle, the PLD inhibitor 1-butanol (1-but) or the inactive inhibitor *t*-butanol (*t*-but) for 15 minutes prior to stimulation with annexin A1 (AnxA1, 10^-9^ M) in **(A)** or with AnxA1 at 10^-10^ M in **(B)**, or carbachol (Cch, 10^-4^ M). Change in peak [Ca^2+^]_I_ is shown in **(A)** Glycoconjugate secretion is shown in **(B)** Data are mean ± SEM from 4 **(A)** or 3 **(B)** independent experiments. *indicates significant difference from basal. ^#^indicates significant difference from either AnxA1 or Cch alone.

Phospholipase D (PLD) is an important regulator of several critical aspects of cell physiology, and is involved in cell functions like endocytosis, exocytosis, cell migration and mitosis (33). We previously showed that the other SPMs such as RvD1, RvE1 and LXA_4_ work through PLD (10–12). To explore if AnxA1 uses PLD, cultured rat conjunctival goblet cells were incubated for 15 minutes with either the PLD inhibitor 1-but or the inactive inhibitor *t*-but, both at 0.3% (**Figure 9A**). AnxA1 (10^-9^ M) alone significantly increased [Ca^2+^]_i_ to a peak of 141.2 ± 50.0 nM (n=4). Neither preincubation with 1-but or *t*-but altered the effect of AnxA1 on the Ca^2+^ response. The increase in [Ca^2+^]_i_ stimulated by the positive control carbachol (10^-4^ M) was significantly blocked by the active inhibitor 1-but, but not the inactive inhibitor *t*-but.

When secretion was measured, AnxA1 (10^-10^ M) alone increased secretion 1.9 ± 0.3 fold above basal (**Figure 9B**, n=3)). Incubation with 1-but, but not t-but, significantly decreased this response that was 0.9 ± 0.1 fold above basal. Similarly to AnxA1, stimulation of secretion by Cch (10^-4^ M) was significantly decreased by 1-but, but not t-but. Based on these findings we conclude that AnxA1 does not active the PLD pathway to stimulate an increase in [Ca^2+^]_i_ but does activate PLD to stimulate secretion in cultured rat conjunctival goblet cells.

### AnxA1 Does Not Increase [Ca^2+^]_i_ But Does Stimulate Secretion Through PLA2 Pathway in Cultured Rat Goblet Cells

**Figure 10:**
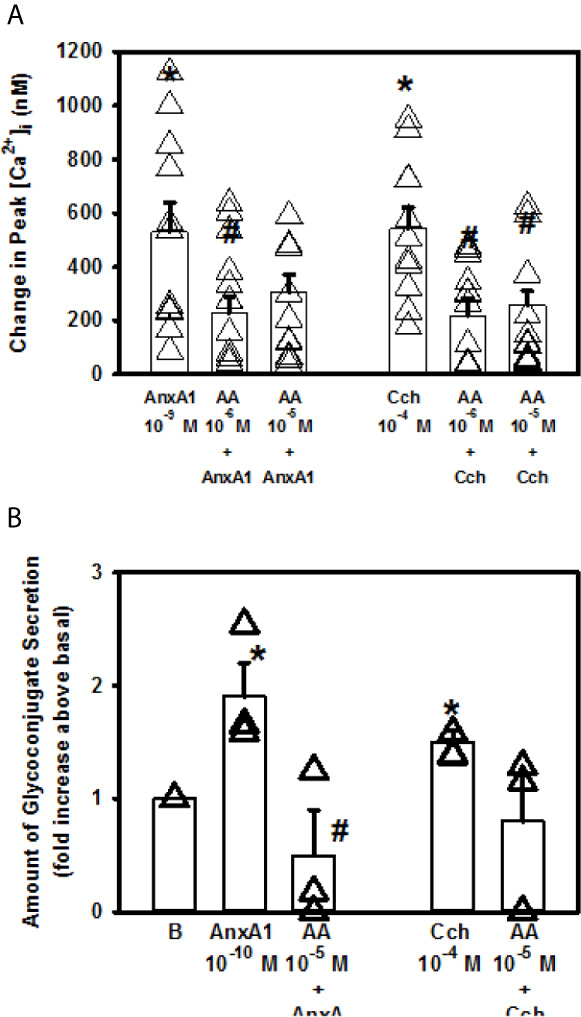
AnxA1 does not active the phospholipase A_2_ (PLA_2_) pathway to increase [Ca^2+^]_i_ but activates it to stimulate secretion in cultured rat conjunctival goblet cells. Rat conjunctival goblet cells were preincubated with either vehicle or the PLA_2_ inhibitor aristolochic acid (AA, 10^-5^ M) for 30 minutes prior to stimulation with annexin A1 (AnxA1, 10^-9^ M) in **(A)** or with AnxA1 at 10^-10^ M in **(B)**, or carbachol (Cch, 10^-4^ M). Change in peak [Ca^2+^]_i_ is shown in **(A)** Glycoconjugate secretion is shown in **(B)** Data are mean ± SEM from 4 **(A)** or 3 **(B)** independent experiments. *indicates significant difference from basal. ^#^indicates significant difference from AnxA1 alone.

Another phospholipase that is present and can be activated in goblet cells is the PLA2 pathway (10–12). To determine if AnxA1 utilizes PLA2 to increase [Ca^2+^]_i_ and secretion, goblet cells were incubated with the PLA2 inhibitor aristolochic acid (AA) for 30 min prior to stimulation with AnxA1 (**Figure 10A**). AnxA1 (10^-9^ M) significantly increased peak [Ca^2+^]_i_ by 530.0 ± 109.2 nM (n=4). Preincubation with AA at 10^-6^ M significantly altered the AnxA1-stimulated response that was 228.1 ± 60.7 nM. AA at 10^-5^ M did not significantly alter the AnxA1 response. The positive control Cch (10^-4^ M)-stimulated response was significantly decreased with AA 10^-6^ and 10^-5^ M from 542.6 ± 78.6 nM to 217.7 ± 65.4 nM and 257.1 ± 52.6 nM, respectively.

AnxA1 (10^-10^ M) increased glycoconjugate secretion by 1.9 ± 0.3 fold above basal (**Figure 10B**, n=3). This response was significantly inhibited by AA (10^-5^ M) to 0.5 ± 0.4. Thus AnxA1 does not active the PLA2 pathway to stimulate an increase in [Ca^2+^]_i_ but does activate PLA2 to stimulate secretion in cultured rat conjunctival goblet cells.

## Discussion

**Figure 11:**
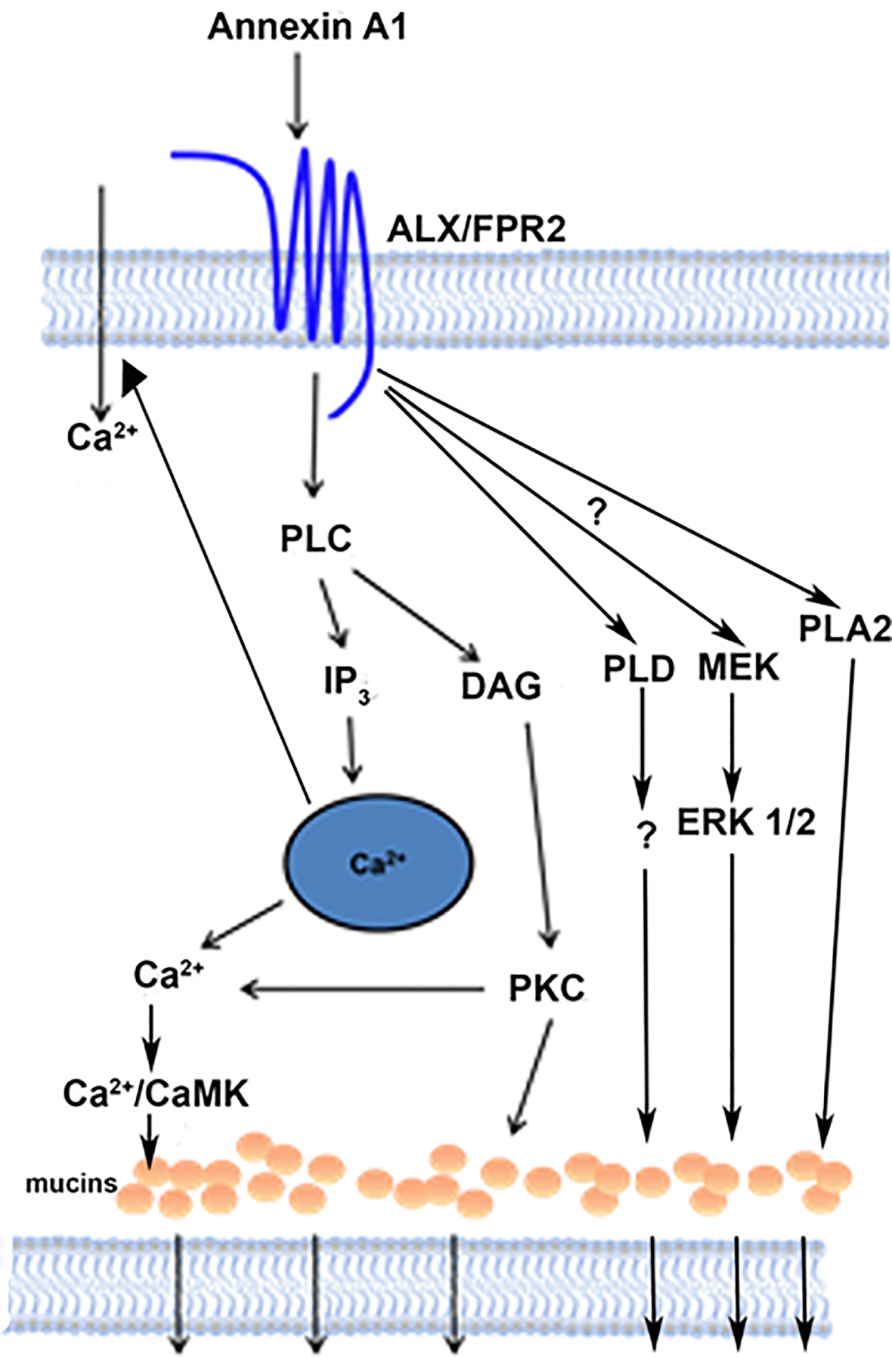
Schematic diagram of signaling pathways activated by AnxA1. AnxA1 binds to the ALX/FPR2 receptor to active the PLC, PLD, and PLA2 pathways, and consequently the secretion of mucins from conjunctival goblet cells. AnxA1 also stimulates influx of Ca^2+^. PLC, phospholipase C; DAG, diacylglycerol; IP_3_, inositol triphosphate; PKC, protein kinase C; Ca^2+^/CamK, calcium/calmodulin-dependent kinase; PLD, phospholipase D; MEK, mitogen-activated protein kinase kinase; ERK 1/2, extracellular regulated kinase; PLA2, phospholipase A2.

In the present study, we showed that AnxA1 contributes to tear film and ocular surface homeostasis by activating the PLC pathway, including its downstream effectors IP_3_ and PKC, through interaction with the ALX/FPR2 receptor (**Figure 11**). This results agrees with findings in Hodges et al. (12) using a desensitization protocol that indicates that AnxA1 interacts with the same receptor as LXA4 and RvD1, the ALX/FPR2 receptor. This interaction increases [Ca^2+^]_i_ and stimulates glycoconjugate secretion including the protective mucin MUC5AC from conjunctival goblet cells. AnxA1-stimulated increase in [Ca^2+^]_i_ is dependent on both intracellular Ca^2+^ stores and influx of extracellular Ca^2+^ consistent with an interaction with Orai and STIM-1 known to regulate these processes in most tissues in which stimulation of G-protein-coupled receptors activates PLC (34).

AnxA1 is present in a variety of other tissues like bone marrow, the intestine and lungs (14, 35). In this study, we showed by RT-PCR that AnxA1 is expressed in conjunctival goblet cells and thus is available to activate these cells. This indicates that AnxA1 not only works during ocular inflammation, but also under physiological conditions, by secreting a basal level of mucins to the tear film and thus maintaining ocular homeostasis. We have demonstrated this for other SPMs including LXA_4_, RvD1, RvD2, maresin 1, and RvE1 (10–12). Unlike the lipid SPMs AnxA1 is a protein, thus we were able to verify its expression by RT-PCR. In contrast, complex lipidomic measurements are needed to detect the presence of the lipid SPMs. As the lipid LXA_4_ is produced by the cornea, it is possible that LXA_4_ could diffuse *via* tears to goblet cells in order to stimulate glycoconjugate secretion. In contrast, AnxA1 is found in conjunctival goblet cells and could be secreted to act upon goblet cells or stratified squamous cells. As AnxA1 is both present and has a role in goblet cell function, AnxA1 likely plays a role in ocular health under physiological conditions as well as preventing or treating the multitude of ocular surface inflammatory diseases. Future study of the regulation of AnxA1 synthesis and secretion in conjunctival goblet cells is warranted.

The ALX/FPR2 receptor is a G-protein-coupled receptor and is the receptor to which AnxA1 binds (36). In addition to its presence on goblet cells (12) the ALX/FPR2 receptor has been identified in human neutrophils, epithelial cells, endothelial cells and monocytes (37, 38). Using ALX/FPR2, AnxA1 functions to resolve diverse inflammatory events, including reduction of joint injury in experimental arthritis (39), lessening of salivary gland inflammation (40), and contributes to corneal wound healing (41). AnxA1 in uterine epithelial cells also activates the ALX/FPR2 receptor to upregulate the membrane-spanning mucin MUC1, tight junction molecules claudin-1 and occludens 1 to increase adherence of trophoblast steroids, a model for embryo implantation (42). Herein we showed that AnxA1 activates the ALX/FPR2 receptor in rat conjunctival goblet cells and that this activation leads to the increase in [Ca^2+^]_i_ and secretion of mucins into the tear film. We previously studied the SPMs LXA_4_ and RvD1, which also activate the ALX/FPR2 receptor in rats. Cooray et al. have shown that the ALX/FPR2 receptors can homodimerize or heterodimerize with FPR1 or FPR3 depending on which agonist is bound (43). In addition, ALX/FPR2 has multiple binding sites notably different lipid and protein sites and binds to multiple compounds (44). AnxA1, a protein, and RvD1 and LXA_4_, which are lipids, bind to different receptor regions in addition to inducing dissimilar receptor conformations (45). This differential binding and a potential difference in FPR receptor family dimerization could explain the complex desensitization we obtained with addition of AnxA1, LXA_4_, and RvD1 (46). These dissimilarities could make the three compounds differ in their effectiveness to treat ocular allergy and other forms of ocular inflammation.

This differential binding of AnxA1, LXA_4_, and RvD1 to bind to the same receptor in conjunctival goblet cells could also lead to ligand-specific activation of signaling pathways. In fact, the AnxA1, LXA_4_, and RvD1 activation of downstream signaling cascades are not fully similar. While AnxA1, LXA_4_, and RvD1 each activate the PLC pathway, only LXA_4_ and RvD1 activate PLD, PLA_2_ and ERK 1/2 (10, 12) to stimulate an increase both in [Ca^2+^]_i_ and mucin secretion. This pattern of pathway activation is consistent with AnxA1 activating the peptide site on ALX/FPR2, whereas LXA_4_ and RvD1 activate the lipid site or overlapping sites.

In the present study, we found AnxA1 to have a peak increase in [Ca^2+^]_i_ at a 10^-9^ M concentration, while the maximum response for secretion was at 10^-10^ M. Furthermore, AnxA1 at 10^-8^ M did not even increase secretion. These findings could be due to several factors. The increase in [Ca^2+^]_i_ occurs very rapidly, within seconds, and is measured in the time frame of seconds. In contrast, secretion, although it can occur rapidly, is measured after 2 hours. The rapid increase in [Ca^2+^]_i_ may not directly link to secretion. The peak [Ca^2+^]_i_ measured is primarily a function of Ca^2+^ release from intracellular stores whereas stimulation of secretion also is regulated by the subsequent influx of Ca^2+^ as well as the size of the intracellular Ca^2+^ stores. The maximum concentration of AnxA1 used to increase the [Ca^2+^]_i_ compared to that used for secretion may result from the differential dependence on the use of distinct mechanisms of Ca^2+^ handling in the goblet cells.

In further support of the independence of AnxA1 concentration on peak [Ca^2+^] and secretion is that secretion, but not peak [Ca^2+^] is stimulated by AnxA1 activation of PLA_2_, PLD, and ERK1/2. Thus the increase in peak [Ca^2+^] and secretion are differentially regulated by AnxA1. This is in contrast to the increase in peak [Ca^2+^] and secretion that when stimulated by LXA_4_ or RvD1 are both dependent on activation of the PLC, PLD, and PLA_2_ pathways. This finding is also consistent with binding of the protein and the lipids to different sites on ALX/FPR2.

In the present study we found that AnxA1 was able to significantly increase [Ca^2+^]_i_ at a notably low concentration (10^-12^ M) in conjunctival goblet cells. In contrast, LXA_4_ increased [Ca^2+^]_i_ at 10^-8^ M (10). Unfortunately the effect of RvD1 concentration on [Ca^2+^]_i_ was not performed at concentrations below 10^-10^ M (unpublished data, DA Dartt 2020). The differential effect of AnxA1 and LXA_4_ concentration on [Ca^2+^]_i_ is consistent with their binding to different areas on the ALX/FPR2 receptor.

Furthermore, we found that AnxA1 was not dependent on either the ERK 1/2, PLA2, or the PLD pathway to increase [Ca^2+^]_i_ although AnxA1 does utilize these pathways to stimulate secretion. In conjunctival goblet cells AnxA1 did not activate ERK 1/2 pathway to increase [Ca^2+^]_i_, but rather more likely AnxA1 increased [Ca^2+^]_i_ to activate ERK1/2. ERK1/2 activity can be Ca^2+^-dependent *via* activation of PLC by a G-protein-coupled receptor or can be independent of Ca^2+^ when activated by β-arrestin-dependent down regulation of G-protein-coupled receptors (47). Published literature showed that in multiple tissues the binding of AnxA1 to the ALX/FPR2 receptor leads to the transient phosphorylation of ERK 1/2 (7, 13, 48, 49), which is associated with a prompt increase in [Ca^2+^]_i_ (7, 38, 50). This suggests that in goblet cells, the activation of ERK 1/2 occurs downstream of the rise of [Ca^2+^]_i_ after activation of PLC. Similarly for AnxA1 activation of PLD, the rise in Ca^2+^ occurs before activation of PLD. An increase in Ca^2+^ and activation of PKC as could occur by PLC stimulation could activate PLD in the goblet cells as documented in a mast cell line (51). The signaling pathways activated by AnxA1 are complex, cell specific, and species dependent.

The function of AnxA1 has been studied in a variety of disease models in order to explore this agonist’s potential as a novel anti-inflammatory therapeutic agent, including models of inflammatory bowel disease (52), rheumatoid arthritis (53), chronic atherogenesis (54), myocardial reperfusion injury (55), myocardial infarction (56), and now ocular inflammation. AnxA1 is thought to have potential as a new treatment option for inflammation by being an effector molecule of glucocorticoids (57). Synthetic glucocorticoids play an extensive and crucial role in the treatment of unwanted and uncontrolled inflammation but come with potentially harmful metabolic side effects with long-term use (57). It is believed that therapeutic application of endogenous anti-inflammatory mediators like AnxA1 will be effective and cause fewer side effects by inducing natural pathways of resolution of inflammation (58–60). AnxA1 could be used therapeutically to treat the multiple inflammatory diseases of the ocular surface especially dry eye and ocular allergy as well as to prevent these diseases or to maintain ocular surface homeostasis in health.

In conclusion, our findings demonstrate that the glucocorticoid effector protein AnxA1 has potential in prevention or as a new treatment for dry eye, allergic conjunctivitis and other forms of ocular surface inflammation through its effect on conjunctival goblet cells. AnxA1 works through activation of the ALX/FPR2 receptor to stimulate multiple signaling pathways, ultimately leading to the secretion of high molecular weight glycoconjugates including mucins into the ocular tear film. These mucins are a crucial component of a healthy tear film, and thus the regulation of mucin secretion from conjunctival goblet cells by AnxA1 is an important contributor to ocular surface health, and prevention of ocular surface disease and treatment of these diseases.

## Author’s Note

This manuscript is dedicated to Robin R. Hodges who passed away on March 13, 2021. For over 30 years she managed the Dartt laboratory ensuring its excellence. She was respected and beloved by all.

## Data Availability Statement

The raw data supporting the conclusions of this article will be made available by the authors, without undue reservation.

## Ethics Statement

The animal study was reviewed and approved by Schepens Eye Research Institute Animal Care and Use of Committee.

## Author Contributions

AL, MO, JB, and RH performed experiments. AL, RH, TU, CS, and DD wrote the manuscript. All authors contributed to the article and approved the submitted version.

## Funding

This work was supported by Norwegian Research Council to AL and by NIH R01 EY019470 to DD and RO1GM038765 to CS.

## Conflict of Interest

The authors declare that the research was conducted in the absence of any commercial or financial relationships that could be construed as a potential conflict of interest.

## Supplementary Material

Supplementary Figure 1 Chelation of $\text{C}{\text{a}}_{i}^{2+}$ blocks AnxA1-stimulated increase in [Ca^2+^]_i_ in cultured rat conjunctival goblet cells. Cultured rat goblet cells were preincubated with the Ca^2+^ chelator BAPTA/AM (10^-4^ M) prior to stimulation with either AnxA1 (10^-9^ M) or carbachol (Cch, 10^-4^ M) and change in peak [Ca^2+^]_i_ shown. Data are mean ± SEM from 4 independent experiments. * indicates significant difference from basal; # indicates significant difference from either AnxA1 or Cch alone.

Supplementary Figure 2 AnxA1 is not dependent on protein kinase A (PKA) to increase [Ca^2+^]_i_ in cultured rat conjunctival goblet cells. Goblet cells were preincubated with the PKA inhibitor H89 for 30 minutes before stimulation with vehicle, annexin A1 (AnxA1, 10^-9^ M) in **(A)** or with AnxA1 (10^-10^ M) in **(B)**, or vasoactive intestinal peptide (VIP, 10^-8^ M). Change in peak [Ca^2+^]_i_ is shown in **(A)**. Glycoconjugate secretion is shown in **(B)**. B indicates basal (vehicle) in **(B)**. Data are mean ± SEM from 9 (A) or 3 **(B)** independent experiments. * indicates significant difference from zero; # indicates significance from AnxA1 or VIP alone.
