# Supplementary figures and images for: Pro-Resolving Mediator Annexin A1 Regulates Intracellular Ca2+ and Mucin Secretion in Cultured Goblet Cells Suggesting a New Use in Inflammatory Conjunctival Diseases
Source: Front Immunol. 2021 Apr 22;12:618653. doi: 10.3389/fimmu.2021.618653 (PMC8100605; doi:10.3389/fimmu.2021.618653)

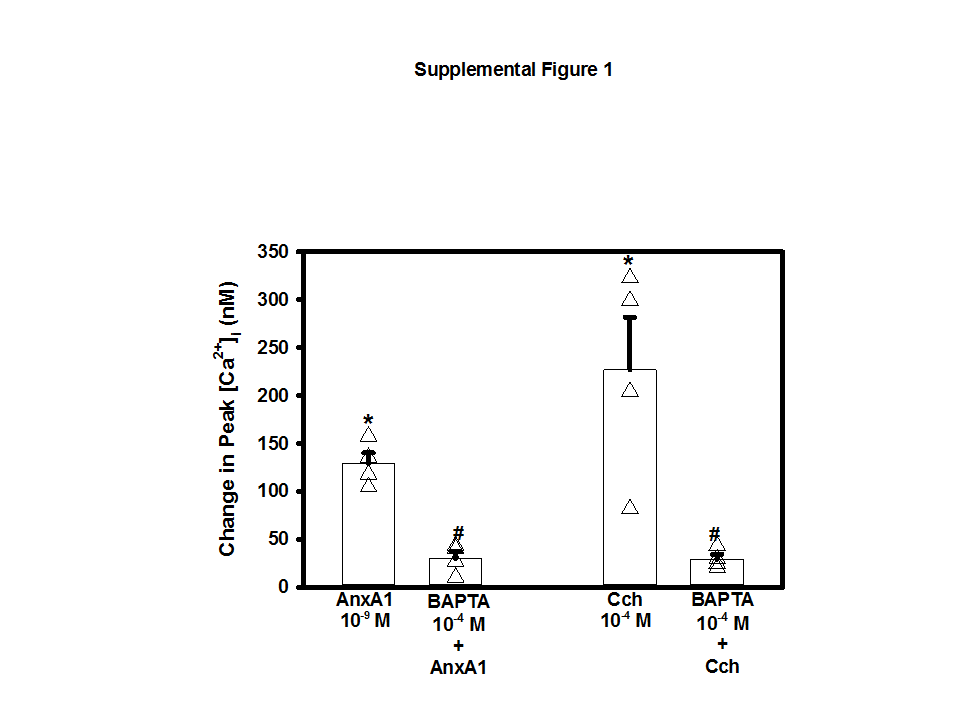

Supplement: Supplementary Figure 1 — Chelation of Cai2+ blocks AnxA1-stimulated increase in [Ca2+]i in cultured rat conjunctival goblet cells. Cultured rat goblet cells were preincubated with the Ca2+ chelator BAPTA/AM (10-4 M) prior to stimulation with either AnxA1 (10-9 M) or carbachol (Cch, 10-4 M) and change in peak [Ca2+]i shown. Data are mean ± SEM from 4 independent experiments. * indicates significant difference from basal; # indicates significant difference from either AnxA1 or Cch alone. [file Image_1.tif]

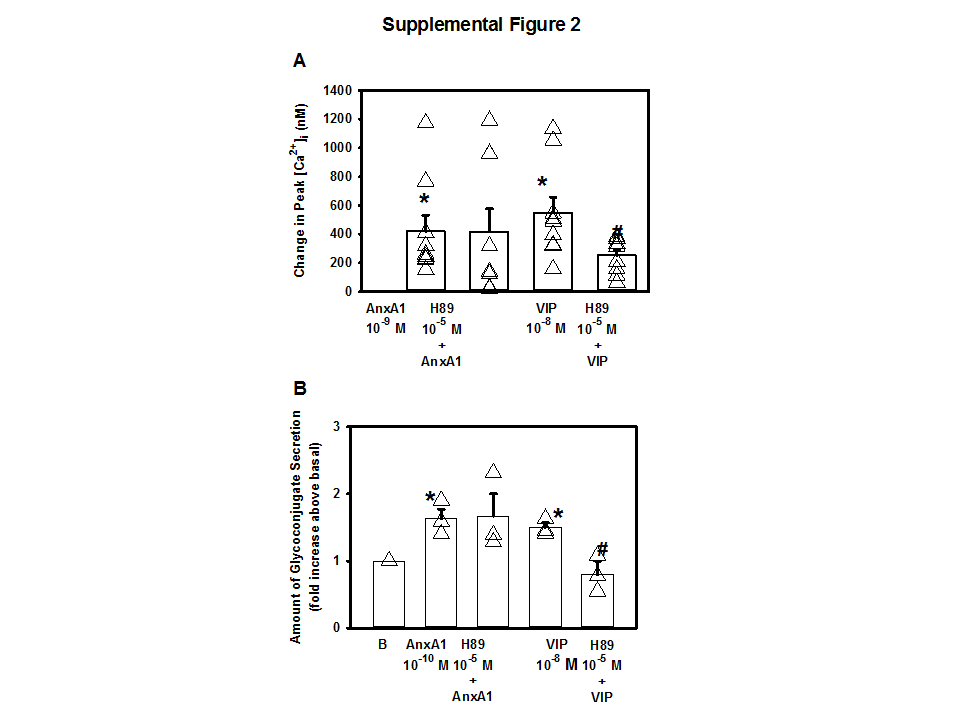

Supplement: Supplementary Figure 2 — AnxA1 is not dependent on protein kinase A (PKA) to increase [Ca2+]i in cultured rat conjunctival goblet cells. Goblet cells were preincubated with the PKA inhibitor H89 for 30 minutes before stimulation with vehicle, annexin A1 (AnxA1, 10-9 M) in (A) or with AnxA1 (10-10 M) in (B), or vasoactive intestinal peptide (VIP, 10-8 M). Change in peak [Ca2+]i is shown in (A). Glycoconjugate secretion is shown in (B). B indicates basal (vehicle) in (B). Data are mean ± SEM from 9 (A) or 3 (B) independent experiments. * indicates significant difference from zero; # indicates significance from AnxA1 or VIP alone. [file Image_2.tif]
